# Universal Thermal Climate Index (UTCI) and adverse pregnancy outcomes in Ahvaz, Iran

**DOI:** 10.1186/s12978-022-01344-7

**Published:** 2022-02-02

**Authors:** Narges Khodadadi, Maryam Dastoorpoor, Narges Khanjani, Afsaneh Ghasemi

**Affiliations:** 1 Department of Epidemiology and Biostatistics, Menopause Andropause Research Center, Ahvaz Jundishapur University of Medical Sciences, Ahvaz, Iran; 2 Environmental Health Engineering Research Center, Kerman University of Medical Sciences, Kerman, Iran; 3 Department of Public Health, School of Public Health, Fasa University of Medical Sciences, Fasa, Iran

**Keywords:** Temperature, Thermal index, Universal Thermal Climate Index, Pregnancy outcome

## Abstract

**Background:**

Climate change may jeopardize the health of mothers and their offspring. There are few studies on the association between increasing temperature and pregnancy outcomes. The aim of this study was to investigate the relation between Universal Thermal Climate Index (UTCI) and adverse pregnancy outcomes including stillbirth, low birth weight (LBW), preterm labor (PTL), spontaneous abortion (SA), preeclampsia and hypertension in Ahvaz, Iran.

**Methods:**

Distributed Lag Non-linear Models (DLNM) combined with quasi-Poisson regression were used to research the effect of UTCI on adverse pregnancy outcomes. The effect of time trend, air pollutants (NO_2_, SO_2_ and PM_10_), and weekdays were adjusted.

**Results:**

The results showed that the low values of UTCI index (11.6 °C, in lags 0–6, 0–13) caused significant increase in the risk of preterm labor. However, hot thermal stress (high UTCI) significantly increased the risk of stillbirth in lag 0–13. We did not observe any significant relation between UTCI and other pregnancy outcomes in this study.

**Conclusions:**

It seems like both hot and cold weathers can be associated with adverse pregnancy outcomes.

## Plain Language Summary

Scientists have found that climate change has adverse effects on human health. Because pregnant women are one of the most vulnerable groups, these negative impacts may affect their fetuses, which make up the next generation. In this study, we examined the effect of temperature on some pregnancy outcomes, including low birth weight, preterm labor, spontaneous abortion, preeclampsia and gestational hypertension in Ahvaz city, which is one of the hottest cities in the world.

One way to assess temperature impact on humans is by using temperature indicators. In this study we used the Universal Thermal Climate Index (UTCI) for this purpose. The data at different time intervals were collected and evaluated with specific models.

Our results showed that low values of UTCI, which is equivalent to cold weather, significantly increase the risk of preterm labor. But, high levels of UTCI, which means hot weather, significantly increase the risk of stillbirth.

In conclusion; both hot and cold weather can be associated with adverse pregnancy outcomes in Ahvaz city. Therefore, pregnant women should protect themselves from exposure to hot and cold weather.

## Background

Scientists believe that the adverse health outcomes related to climate change are increasing rapidly [1]. Pregnant mothers and growing embryos are among the populations most vulnerable to the effects of climate change [2]. However, the effect of increasing temperature on reproductive outcomes is not thoroughly understood [2, 3]. Some studies have reported a possible relation between preterm birth, stillbirth, and low birth weight with ambient temperature [4]. For example, Yu et al. reported the effect of extreme weather on preterm birth in the tropical island of Puerto Rico [5]. Apparently, increase in temperature in the warm season is significantly more dangerous than the cold season [6], and studies have reported a positive relation between preterm birth and heat wave exposure during pregnancy [7, 8].

There are also several studies which have showed connections between stillbirth and high temperatures during the week before delivery [9–12] or during early pregnancy [7]. In Sweden, Hartig et al. found that the risk of LBW < 1500 g for male infants increases in the summers with cold weather, which is probably due to the stress caused by the unpleasant weather [13]. Exposure to extreme temperatures in different months of pregnancy may have different results. In a 2017 analysis in the US the risk of low birth weight increased for cold exposures during the second and third trimesters, and for hot exposures during the third trimester of pregnancy [10]. Another study found that women with their first pregnancy in the summer [14] or those exposed to high temperatures during the first trimester of pregnancy, as well as 30 days after pregnancy, had a higher risk for preeclampsia [15]. The relation between gestational hypertension as one of the adverse consequences of pregnancy and temperature in both cold and hot periods has been investigated in several studies, and the incidence was mostly higher in winter [16–19]. Interest in evaluating environmental factors as a determinant of adverse birth outcomes has increased, and one method of investigating this issue is through temperature indices [20].

Since 1950, human thermal comfort in both indoor and outdoor environments has been evaluated in multiple research, leading to various numerical and diagram-based indices [21]. One of the most popular indices used to measure thermal stress in outdoor spaces is the Universal Thermal Climate Index (UTCI) which was proposed more than 10 years ago by the International Society for Biomarkers (ISB) [21]. It is used in key applications such as daily forecasting and alerting, urban and regional planning, environmental epidemiology, and climate impact research across all climates. The purpose of UTCI is to evaluate outdoor thermal conditions, in the main fields of human biometrics, in terms of a one-dimensional quantification that summarizes the interaction of ambient temperature, wind speed, humidity, and long and short-wave heat fluxes [22]. UTCI is defined as the equivalent ambient temperature (°C) of a reference environment that provides the physiological response of a reference person, in the real environment [23]. The multi-node model of human thermoregulation is used to calculate the physiological response to meteorological inputs [24].

As global warming continues, the incidence of significant temperature changes are more likely. The aim of the this paper was to study the relation between UTCI and stillbirth, low birth weight (LBW), preterm labor (PTL), spontaneous abortion (SA), preeclampsia and gestational hypertension in Ahvaz, Iran.

## Methods

### Study site

Ahvaz is the seventh most populous city of Iran and the capital city of Khuzestan province in the south-west of Iran. Ahvaz is located at 31° 20′ N and 48° 40′ E. It’s area is185 km^2^ and it is 12 m above sea level. In the 2016 census, the population of this city was approximately 1,300,000 (Iran Statistics center, 2016). Ahvaz has a desert climate with hot long summers and short mild winters. Ahvaz is often the hottest city in the world during summer, with high consistent temperature between 45 and 50 °C. The annual average temperature in this city is 25.4 °C. Ahvaz recorded the temperature of 54 °C which was the highest temperature in the world on June 29, 2017 [25].

### Data

#### Study population

Data about adverse pregnancy outcomes including stillbirth, LBW, PTL, SA, preeclampsia and hypertension (diagnosed in the clinic and recorded with date in the patients’ files) were collected from the population of pregnant women that visited two big referral hospitals in Ahvaz(the Imam Khomeini, and Razi Hospital), from 2008 to 2018 (10 years). The diagnoses were based on ICD-10 and included the following codes; stillbirth (Z37.1), low birth weight (P07.0), preterm labor (O60), spontaneous abortion (O03), pre-eclampsia (O14), and gestational hypertension (O13).

The data was inquired on a daily basis, from the beginning of April 2008 until March 2018. The total number of pregnant women who visited the Obstetrics and Gynecology department during this 10-year period was 150,766.

#### Meteorological data

Meteorological parameters including average daily temperature, maximum daily temperature, minimum daily temperature, average wind speed, relative humidity, and cloudiness, from March 20, 2008 to March 20, 2018 were obtained from the Khuzestan Meteorological Department. Ahvaz city has one synoptic meteorology station, in which different atmospheric factors such as temperature, type of clouds, rainfall and cloudiness is determined, and recorded at certain hours, daily. This station is 22.5 m above sea level, at 48° 40′ E longitude and 31° 20′ N latitude, inside Ahvaz.

Data about ambient air pollutants were inquired from the Ahvaz Environmental Protection Organization and included SO_2_, PM_10_, NO_2_. There are four air pollution monitoring stations in Ahvaz city and in this study the average of the four stations were used.

#### Missing data estimation

Missing air pollutant data were estimated using the EM (Expectation–Maximization) method [26]. The Expectation Maximum method uses the available data to create regression models that estimate missing data. In this method, regression parameters are re-estimated several times and updated regularly using new sets. That is, initially the available data is used to estimate the parameters of the model. Then the available data and the estimated data, are used to re-estimate the missing data. This process is repeated until the difference between two consecutive regression coefficients becomes less than 10^–6^ [26]. In this study, there was no missing meteorological data, but there was less than 10% missing in air pollution data, that was estimated using EM.

### UTCI Index

The UTCI index is calculated based on albedo of skin (%), albedo of ground (%), vapour pressure (hPa), relative humidity (%), dew point temperature (°C), sun altitude (degree), clothing insulation (clo), diffuse solar radiation (W/m^2^), direct solar radiation on horizontal plane (W/m^2^), global solar radiation on horizontal plane (W/m^2^), reflected solar radiation (W/m^2^), metabolic heat production (W/m^2^), mean radiant temperature (°C), cloudiness (octants), atmospheric pressure (hPa), gender, air temperature (°C), ground temperature (°C), skin temperature (°C), wind speed (m/s) and human movement (m/s). In the lack of access to some of these data, they can be estimated from other data [27]. In order to calculate the UTCI index, some parameters were considered the same for all people. Thus, albedo of skin was assumed to be 30%, albedo of ground 17%, metabolic heat production: 135 W/m^2^, atmospheric pressure: 1000 hPa, gender: female and human movement: 1.1 m/s [27].

Analysis of UTCI index was performed using the RayMan software. The RayMan software is a simulation tool used in human-biometeorology. Details about this software can be found elsewhere [28]. The classification of UTCI index in Iran is presented in Table 1. The thermal comfort range for UTCI is from 9 to 26 °C, this means that in this range there is no thermal stress imposed on humans [29].

**Table 1:** Thermal sensation and different groups of UTCI

UTCI (°C) in Iran*	Thermal sensation	Physiological stress level
< − 40	Very cold	Extreme cold stress
− 27 to − 40	Cold	Very strong cold stress
− 13 to − 27	Cool	Strong cold stress
0 to − 13	Slightly cool	Moderate cold stress
0 to 9	Comfortable	Slight cold stress
9 to 26	Slightly warm	No thermal stress
26 to 32	Warm	Moderate heat stress
32 to 38	Hot	Strong heat stress
38 to 46	Very hot	Very strong heat stress
> 46		Extreme heat stress

In order to investigate the effect of UTCI index on study outcomes, two separate analyses were performed. First, the association between low values (comparison of 1, 5 and 25th percentiles relative to no thermal stress) and high values (comparison of 90, 95 and 99th percentiles relative to no thermal stress) of UTCI index, with the risk of adverse pregnancy outcomes, in zero cumulative models of 0–2, 0–6, 0–13 and 0–21 days lag was determined. The median of UTCI, defined as the no thermal stress class, was 17.5 °C and was used as the basis for comparison with other high and low thermal stress values. Zero cumulative models used in this study were models estimating the effect of exposure from day 0 until a particular day. For example, the 0–2 model, means the effect observed from day 0 (same day, lag = 0) until day 2 (lag = 2 days).

Second, the association between cold thermal stress (comparison of 1th percentile relative to 25th percentile) and hot thermal stress (comparison of 75th percentile relative to 99th percentile) of UTCI index with risk of adverse pregnancy outcomes, in zero cumulative models, 0–2, 0–6, 0–13 and 0–21 days lag was calculated according to methods used in previous references [30–32].

### Statistical analysis

In order to investigate the effect of UTCI on adverse pregnancy outcomes, Distributed Lag Non-linear Models (DLNM) combined with quasi-Poisson regression models were used. The DLNM model, is based on cross-basis functions, and is used for simultaneous estimation of the nonlinear relation between exposure and outcome in different time lags [33].

In this study, a natural cubic-spline DLNM was used to determine the nonlinear relation of UTCI index and also the cumulative lag effects up to a maximum of 21 days, with adverse pregnancy outcomes.

Spline knots were set at equally spaced values on the log scale of lags. The long term, seasonal trend of adverse pregnancy outcomes was moderated by a natural cubic spline function of time with 7 degrees of freedom per years of study (10 years). PM_10_, SO_2_and NO_2_ were controlled using the stratified distributed lag model for up to 7 days lag with 3 degrees of freedom [30]. Also, the variable of holidays and weekdays was adjusted as a categorical variable in the final model [30]. Akaike Information Criterion (AIC) models were used to select the most appropriate model and degrees of freedom (knots) for thermal index and lags [33]. Three degrees of freedom were considered as the best model for thermal index and time lags. The risk ratio and 95% confidence interval were estimated for the associations. The analysis was performed utilizing R software version 3.5.3 through the dlnm package. P-values < 0.05 were considered significant.

## Results

Descriptive index of UTCI, stillbirth, LBW, PTL, SA, preeclampsia and gestational hypertension are presented in Table 2. During the 10-year study period, the highest and lowest adverse pregnancy outcomes were respectively preterm labors (5776 cases) and stillbirths (1965 cases). The mean ± SD of UTCI was 29.0 ± 10.1 (Table 2).

**Table 2:** Descriptive statistics of adverse pregnancy outcomes, air pollutants and UTCI in Ahvaz city, 2008–2018

Variable (mean per day)	N	Mean ± SD	Median	Min	Max
Stillbirth	1965	0.5 ± 0.8	0	0	5
LBW^a^	863	0.2 ± 0.6	0	0	5
PTL^b^	5776	1.6 ± 1.9	1	0	12
SA^c^	5063	1.4 ± 1.4	1	0	8
Pre-eclampsia	4357	1.2 ± 1.2	1	0	8
Gestational hypertension	4030	1.1 ± 1.2	1	0	13
NO_2_^d^ (µg/m^3^)	–	46.4 ± 43.1	35.6	1.5	443.8
SO_2_^e^ (µg/m^3^)	–	48.8 ± 57.0	35.9	0	907.4
PM_10_^f^ (µg/m^3^)	–	216.9 ± 278.3	149.2	1.8	4324.2
UTCI^g^	–	29.0 ± 10.1	28.6	5.8	49.8

^a^Low birth weight

^b^Preterm labor

^c^Spontaneous abortion

^d^Nitrogen dioxide

^e^Sulfur dioxide

^f^Particulate matter less than 10 microns

^g^Universal thermal climate index

### UTCI index and adverse pregnancy outcomes

The results of Table 3 shows that the low value of UTCI index at the 1st percentile compared to no thermal stress, in lags 0–6 and 0–13 significantly increased the risk of preterm labor.

**Table 3:** The cumulative relative risks of adverse pregnancy outcomes in high and low UTCI^a^ values relative to UTCI = 17.5 °C

Outcomes	UTCI value (°C)		Lag 0	Lag 0–2	Lag 0–6	Lag 0–13	Lag 0–21
Stillbirth	H^b^	46.4	1.780 (0.793–3.997)	1.200 (0.521–2.763)	1.725 (0.609–4.890)	2.148 (0.529–8.719)	1.911 (0.283–12.913)
		44.6	1.506 (0.721–3.145)	1.190 (0.559–2.536)	1.542 (0.588–4.044)	1.811 (0.480–6.838)	1.627 (0.263–10.076)
		43.1	1.340(0.667–2.693)	1.179 (0.577–2.408)	1.414 (0.564–3.548)	1.579 (0.439–5.676)	1.411 (0.242–8.238)
	L^c^	16.2	1.008 (0.956–1.064)	0.976 (0.927–1.028)	0.957 (0.899–1.019)	0.946 (0.871–1.026)	0.949 (0.848–1.061)
		14.4	1.004 (0.882–1.142)	0.940 (0.834–1.059)	0.898 (0.776–1.039)	0.890 (0.731–1.082)	0.874 (0.664–1.150)
		11.6	0.949 (0.694–1.297)	0.880 (0.657–1.178)	0.814 (0.562–1.180)	0.858 (0.515–1.428)	0.769 (0.376–1.573)
LBW	H	46.4	0.623 (0.164–2.368)	0.990 (0.268–3.658)	2.505 (0.488–12.871)	0.781 (0.082–7.403)	0.111 (0.005–2.342)
		44.6	0.647 (0.193–2.169)	1.209 (0.367–3.984)	2.635 (0.577–12.028)	0.959 (0.112–8.225)	0.168 (0.009–3.175)
		43.1	0.665 (0.212–2.091)	1.361 (0.438–4.231)	2.641 (0.618–11.284)	1.056 (0.131–8.507)	0.217 (0.012–3.850)
	L	16.2	1.004 (0.922–1.093)	0.998 (0.921–1.082)	0.96 (0.868–1.062)	0.977 (0.852–1.12)	0.935 (0.773–1.132)
		14.4	0.954 (0.768–1.186)	0.986 (0.811–1.201)	0.869 (0.679–1.112)	0.833 (0.589–1.177)	0.763 (0.476–1.223)
		11.6	0.763 (0.430–1.356)	0.953 (0.575–1.578)	0.672 (0.349–1.292)	0.475 (0.186–1.211)	0.434 (0.128–1.474)
PTL	H	46.4	0.988 (0.601–1.624)	1.108 (0.656–1.872)	1.192 (0.617–2.303)	1.012 (0.412–2.484)	2.392 (0.699–8.193)
		44.6	1.058 (0.669–1.674)	1.192 (0.737–1.926)	1.285 (0.697–2.368)	1.078 (0.465–2.502)	2.387 (0.748–7.616)
		43.1	1.103 (0.712–1.709)	1.245 (0.789–1.965)	1.343 (0.749–2.409)	1.115 (0.500–2.489)	2.330 (0.767–7.079)
	L	16.2	0.996 (0.964–1.029)	0.977 (0.947–1.007)	0.985 (0.95–1.022)	1.017 (0.97–1.067)	1.014 (0.949–1.082)
		14.4	1.009 (0.936–1.089)	0.980 (0.915–1.049)	1.033 (0.950–1.124)	1.101 (0.977–1.240)	1.108 (0.939–1.307)
		11.6	1.087 (0.910–1.298)	1.086 (0.921–1.282)	**1.337 (1.074–1.664)**	**1.449 (1.042–2.015)**	1.536 (0.973–2.425)
SA	H	46.4	1.019 (0.584–1.780)	0.997 (0.568–1.752)	0.764 (0.378–1.543)	0.738 (0.285–1.910)	0.723 (0.199–2.624)
		44.6	0.942 (0.570–1.556)	1.026 (0.618–1.704)	0.781 (0.409–1.494)	0.719 (0.292–1.769)	0.708 (0.207–2.417)
		43.1	0.892 (0.556–1.432)	1.043 (0.647–1.682)	0.797 (0.430–1.479)	0.713 (0.299–1.700)	0.707 (0.215–2.322)
	L	16.2	1.006 (0.971–1.042)	1.009 (0.975–1.043)	1.005 (0.964–1.047)	1.009 (0.955–1.066)	1.036 (0.961–1.116)
		14.4	0.998 (0.919–1.084)	1.013 (0.939–1.093)	1.001 (0.912–1.098)	1.035 (0.913–1.173)	1.094 (0.918–1.303)
		11.6	0.942 (0.776–1.144)	0.999 (0.836–1.193)	0.966 (0.769–1.213)	1.110 (0.811–1.520)	1.201 (0.774–1.864)
Pre-eclampsia	H	46.4	0.805 (0.458–1.415)	0.770 (0.432–1.370)	1.086 (0.530–2.225)	1.385 (0.529–3.629)	1.166 (0.316–4.303)
		44.6	0.791 (0.475–1.317)	0.772 (0.459–1.298)	1.071 (0.552–2.075)	1.378 (0.555–3.417)	1.126 (0.325–3.895)
		43.1	0.779 (0.481–1.260)	0.774 (0.474–1.264)	1.058 (0.563–1.987)	1.354 (0.566–3.239)	1.073 (0.324–3.557)
	L	16.2	1.028 (0.991–1.066)	1.01 (0.976–1.046)	0.998 (0.958–1.04)	1.011 (0.958–1.067)	0.998 (0.927–1.075)
		14.4	1.052 (0.966–1.146)	1.033 (0.956–1.116)	1.038 (0.946–1.139)	1.062 (0.935–1.207)	0.982 (0.818–1.180)
		11.6	1.050 (0.857–1.285)	1.091 (0.909–1.311)	1.236 (0.981–1.557)	1.261 (0.900–1.766)	0.936 (0.573–1.530)
Gestational hypertension	H	46.4	1.411 (0.758–2.626)	1.023 (0.549–1.908)	1.351 (0.619–2.948)	1.183 (0.411–3.409)	1.045 (0.249–4.387)
		44.6	1.410 (0.805–2.470)	1.037 (0.589–1.824)	1.455 (0.706–2.999)	1.381 (0.506–3.767)	1.151 (0.292–4.541)
		43.1	1.412 (0.832–2.396)	1.052 (0.617–1.795)	1.544 (0.775–3.079)	1.551 (0.590–4.080)	1.248 (0.330–4.717)
	L	16.2	0.968 (0.931–1.006)	0.995 (0.959–1.031)	0.955 (0.913–0.999)	0.962 (0.907–1.021)	0.961 (0.885–1.043)
		14.4	0.932 (0.852–1.020)	0.983 (0.906–1.067)	0.924 (0.834–1.024)	0.963 (0.837–1.109)	0.951 (0.779–1.160)
		11.6	0.897 (0.725–1.109)	0.952 (0.778–1.165)	0.950 (0.731–1.234)	1.110 (0.771–1.600)	1.047 (0.621–1.764)

Statistically significant values are given in bold

^a^Universal Thermal Climate Index (°C)

^b^High UTCI values

^c^Low UTCI values

**Fig. 1:**
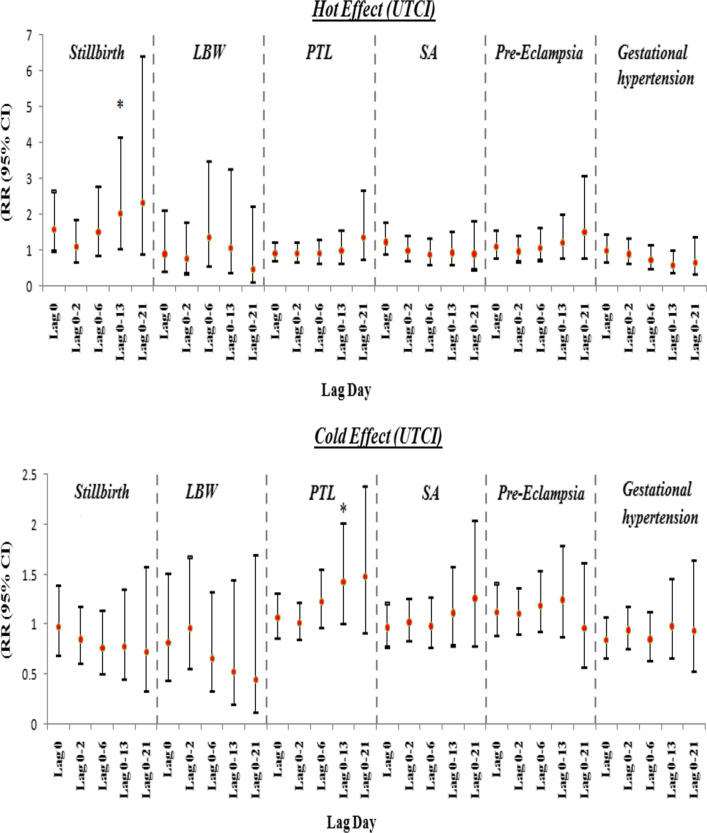
The relative risks (95% CIs) of hot and cold thermal stress of UTCI on adverse pregnancy outcomes at different lag days. *Statistically significant

Also in the intensified thermal stress analysis, cold thermal stress significantly increased the risk of PTL, in the 0–13 lagtime. Finally, hot thermal stress increased the risk of stillbirth in lag 0–13, significantly (Table 4, Fig. 1).

**Table 4:** The cumulative relative risks of adverse pregnancy outcomes in intensified thermal stress of UTCI

	Lag 0	Lag 0–2	Lag 0–6	Lag 0–13	Lag 0–21
Hot effect (UTCI)^a^					
Stillbirth	1.591 (0.957–2.647)	1.087 (0.636–1.858)	1.513 (0.825–2.773)	**2.049 (1.012–4.151)**	2.336 (0.853–6.400)
LBW	0.892 (0.378–2.108)	0.769 (0.335–1.766)	1.369 (0.538–3.485)	1.074 (0.355–3.253)	0.464 (0.097–2.218)
PTL	0.902 (0.672–1.211)	0.897 (0.650–1.238)	0.907 (0.626–1.313)	0.972 (0.613–1.542)	1.376 (0.711–2.665)
SA	1.231 (0.859–1.766)	0.973 (0.673–1.409)	0.887 (0.586–1.343)	0.932 (0.573–1.516)	0.895 (0.443–1.809)
Pre-eclampsia	1.087 (0.759–1.558)	0.965 (0.665–1.402)	1.064 (0.701–1.615)	1.219 (0.742–2.005)	1.524 (0.754–3.081)
Gestational hypertension	0.979 (0.657–1.456)	0.893 (0.600–1.331)	0.731 (0.466–1.145)	0.585 (0.342–1.002)	0.640 (0.301–1.360)
Cold effect (UTCI)^b^					
Stillbirth	0.973 (0.684–1.382)	0.851 (0.615–1.176)	0.761 (0.509–1.138)	0.778 (0.449–1.347)	0.724 (0.334–1.567)
LBW	0.812 (0.437–1.510)	0.965 (0.557–1.671)	0.659 (0.327–1.325)	0.529 (0.195–1.432)	0.448 (0.119–1.692)
PTL	1.064 (0.868–1.303)	1.014 (0.845–1.218)	1.229 (0.974–1.550)	**1.422 (1.008–2.006)**	1.475 (0.914–2.380)
SA	0.968 (0.776–1.207)	1.022 (0.836–1.249)	0.983 (0.765–1.264)	1.112 (0.789–1.568)	1.260 (0.778–2.039)
Pre-eclampsia	1.118 (0.889–1.406)	1.1050 (0.900–1.357)	1.187 (0.923–1.526)	1.248 (0.872–1.788)	0.959 (0.569–1.616)
Gestational hypertension	0.840 (0.660–1.068)	0.943 (0.755–1.177)	0.848 (0.639–1.124)	0.981 (0.663–1.453)	0.932 (0.532–1.634)

Statistically significant values are given in bold

^a^The cumulative effects of hot thermal stress on adverse pregnancy outcomes, with 99th percentile of UTCI (46.4 °C) relative to 75th percentile of UTCI (38.0 °C)

^b^The cumulative effects of cold thermal stress on adverse pregnancy outcomes, with 1st percentile of UTCI (11.6 °C) relative to 25th percentile of UTCI (19.8 °C)

## Discussion

According to the World Health Organization and the Lancet Countdown, one of the biggest consequences of climate change is its risk to human health [34, 35]. The effects of climate change on women’s health has been mentioned in some references [36, 37]. These adverse effects are more pronounced in high-risk populations such as pregnant women and their developing fetuses [37].

In the present study, the results showed that hot thermal stress in the UTCI index, increased the risk of stillbirth. Similar results have been observed in other studies, such as Kanner et al. [39], who studied 11,2005 deliveries between 2002 and 2010 and assessed the association of stillbirth with acute changes in ambient temperature in a low-risk population in the US. These researchers stated that every 1 °C raise in ambient temperature in the week before delivery, increased the risk of stillbirth by 7% [37]. In the warm weather of the Ghanaian tropics, this increase was 12–15% per 1 °C increase in wet-bulb globe temperature (WBGT) [38]. Another study in California found that for every 10 °F increase in apparent temperature in the warm season, the risk of stillbirth increased by 10.4% after 2–6 days of delay [38], and Rammah et al. who studied 708 women, in the warm seasons of 2008–2013 in Harris County, Texas, reported a much stronger effect, and stated that each 10 °F increase in apparent temperature in the week before delivery caused a 45% increase in stillbirth [9]. There are other studies that have reported the effect of increased temperature in 1 week before delivery on stillbirth [39, 40] as well. But one study conducted in Brisbane, Australia, during 2005–2009, stated in addition to the last week of pregnancy, heat stress should also be considered in longer periods, because their results showed that mothers’ exposure to high ambient temperatures in the last 4 weeks of pregnancy increased the risk of stillbirth compared to mothers who were only exposed in the previous week [20]. Systematic reviews conducted in the US and China confirm the positive association between climate change and stillbirth [7, 41, 42]. But in contrary to these findings, in a cohort study from Sweden, hot weather was not associated with an increased risk of stillbirth, and in fact for every 1 °C increase in temperature during pregnancy, the risk of stillbirth decreased by 8% [43]. The reason for these different results, may be due to the differences in climate between Ahvaz (often hot weather) and Sweden (often cold weather), and the fact that people in Sweden face milder heat.

Although the mechanism of the effect of temperature on stillbirth is not yet clear, reduction of amniotic fluid volume, placental damage, or temperature-induced uterine contractions are possible mechanisms [44, 45]. Heat stress can cause a decrease of water and blood volume in the mother’s body, and this may lead to uterine contractions [46]. Also in heat stress, in order to decrease body heat, blood flow to the periphery increases, and this decreases the perfusion of the placental and umbilical blood [47]. These changes can affect blood pressure and viscosity and ultimately impair the growth and survival of the fetus [48].

Concerning preterm labor, the results of this study indicated that low levels of UTCI and cold thermal stress cause a significant increase in the risk of preterm labor. In support of these results, the results of a study by Bruckner et al. in Uppsala, Sweden, showed that extreme cold increases the risk of preterm labor [43]. Also cohort studies conducted in the US [10] and in Shenzhen, China [49], showed that short-term exposure to cold conditions before delivery increased the risk of preterm birth. However, another study that looked at birth outcomes of 1,020,471 pregnant women from 132 cities in China, reported cold exposure (the 95th percentile) in hot areas reduced the risk of preterm birth; and the protective effect was seen especially in the last 3 months of pregnancy (OR 0.784, 95% CI 0.734–0.832) [50].

Some studies have stated that both hot and cold weather, affect PTL. For example, a study from Guangzhou, China reported that extreme heat and extreme cold during the last 4 weeks of pregnancy increased the risk of preterm birth by 17.9% and 10%, respectively [51]. Likewise, in Sabzevar, Iran, both very hot and very cold temperatures were associated with an increase in PTL [52]. Li et al. [53], in Brisbane, showed that during the years under study (1993–2013), the effect of low temperature was stronger than the effect of high temperature for preterm delivery [53]. However, the results of Basu et al. [12], from California, on approximately 60,000 newborns, showed that high ambient temperature was significantly associated with preterm labor for all mothers, without considering mothers’ racial or ethnic group, age, education, or baby gender. The results showed that for every 10° F (5.6 °C) increase in weekly mean apparent temperature in lag 6, the risk of preterm labor increased by 8.6% (95% CI 6.0–11.3%) [12].

The mechanism of the effect of cold air on preterm labor may be related to the effect of cold stress. Experimental research on mammals show that cold weather increases the levels of stress hormones [54, 55] and the transfer of these hormones from mother to fetus may cause earlier birth [56, 57]. Researchers think cold stress increases the level of corticosteroids, and this may accelerate parturition and lead to preterm labor [58]. Increased levels of corticosteroids also suppress the immune system, therefore latent infections are activated and as a result, the risk of preterm labor increases [59].

In terms of other adverse pregnancy outcomes, the results of our study did not show any significant relation between the effect of UTC Index and LBW, SA, preeclampsia and gestational hypertension. In line with our results, the Bruckner study in Sweden (2014) also found no significant relation between ambient temperature and birth weight [43]. But some other articles have observed relations between the effect of ambient temperature (hot weather and cold weather) and these outcomes. For example, in a recent systematic review including studies conducted in the US, the highest association between climate change and adverse pregnancy outcomes was reported for LBW [41]. Also a large 20-year study in 19 African countries found that the risk of LBW increased significantly, with increased number of hot days [60]. Other studies have reported a positive association between LBW risk and hot weather in the United States [61, 62] and cold weather in Sweden [13].

Recently, Sun et al. conducted a case–control study in Guangzhou, China, and found a positive relation between exposure to moderate or higher heat during pregnancy and the risk of miscarriage [63].

In the case of gestational hypertension, many studies have observed an increased risk in cold weather [16, 18, 19]. In Brazil, Melo et al. observed that gestational hypertension increased significantly during the colder months, especially in August when the southern hemisphere is in winter [17]. A recent study from China found that exposure to very cold weather before pregnancy increased the risk of preeclampsia, eclampsia and gestational hypertension; and conversely, exposure to cold in the first half of pregnancy reduces, while hot weather increases the risk of hypertensive disorders in pregnancy [64]. But, Elongi et al. found no association between gestational hypertension and seasonal changes, during their 36-month study, in Mississippi [65], which is consistent with the results of the present study. However, Auger et al. conducted a study in Quebec, Canada, and noticed that hot weather during pregnancy increased the risk of preeclampsia, while cold weather increased this risk, only at the end of pregnancy [66]. A study of all deliveries in the Yvelines region of France, between 2008 and 2011 found that high temperature was associated with an increase in severe preeclampsia and the risk was higher when the fetus was conceived in summer than in winter [15]. The reason for the discrepancy between study results may be due to differences in demographic characteristics, adaptation, and seasonal or climate effects [10, 40]. Also, the different study designs incorporated further complicate the comparison of results [40].

## Strengths and limitations

This was the first study conducted on the relation between the UTCI index and adverse pregnancy outcomes in the world. The results of this study can help in clarifying the effect of climate change on adverse pregnancy outcomes. One of the limitations of this study was that it did not take into account individual characteristics such as living conditions, activity during pregnancy, and clinical reasons which might have been confounders. Meanwhile, we did not have individual data and we could not adjust for these variables. Therefore, the results should be interpreted with caution.

## Conclusions

Overall, our results showed that heat stress measured according to UTCI index was associated with an increased risk of stillbirth, while low levels of UTCI and cold heat stress were associated with increased risk of PTL. But, we did not find a significant relation between the UTCI index with other adverse pregnancy outcomes. The results of this study and similar studies can help promote the general health of pregnant women, raise awareness about the risk of temperature stress, and help reduce the adverse effects of temperature on the next generation.

## Data Availability

Anonymous, group level data is available by communication with the corresponding author.

## Abbreviations

UTCI: Universal Thermal Climate Index
DLNM: Distributed Lag Non-linear Models
NO_2_: Nitrogen dioxide
SO_2_: Sulfur dioxide
PM_10_: Particles with a diameter of less than 10 µm
EM: Expectation Maximization
AIC: Akaike Information Criteria
